# Building the Infrastructure for Sustainable Digital Mental Health: It Is “Prime Time” for Implementation Science

**DOI:** 10.2196/78791

**Published:** 2025-07-21

**Authors:** Gillian Strudwick, Iman Kassam, John Torous, Sean Patenaude

**Affiliations:** 1 Campbell Family Mental Health Research Institute, Centre for Addiction and Mental Health, 1001 Queen St West, Toronto, ON, M6J 1H1, Canada, 1 4165358501; 2 Division of Digital Psychiatry, Beth Israel Deaconess Medical Center, Boston, MA, United States

**Keywords:** mental health, psychiatry, implementation science, technology, digital health, innovation

## Abstract

Despite the growing enthusiasm for and a proliferation of digital mental health innovations, their integration into routine clinical care remains limited—often stalled at the pilot, research, or demonstration stage. This editorial argues that the successful adoption, scale-up, and sustainability of digital mental health innovations require intentional infrastructure, not just technology. Using the Non-Adoption, Abandonment, Scale-Up, Spread, and Sustainability (NASSS) implementation science framework, we examine how challenges across the seven framework domains (condition, technology, value proposition, adopters, organization, wider context, and their interactions over time) continue to hinder meaningful progress. We describe a focused digital mental health innovation infrastructure as a model for overcoming these barriers. Drawing on experiences from the Digital Innovation Hub at Canada’s largest mental health and addictions teaching hospital, we illustrate how investing in the right infrastructure may move digital mental health innovations from “promising” to “impactful.” We call for global collaboration to share knowledge and accelerate the real-world integration of digital innovations in routine mental health clinical care.

## Introduction

Digital mental health innovations have rapidly transitioned from the sidelines to a core area of innovation in mental health care [1,2]. Across clinical practice, research, and the technology industry, interest in using digital mental health innovations has increased significantly [3-5]. These innovations hold promise in addressing long-standing and emerging challenges in mental health clinical care that persist around the world, such as limited access to care, health human resource challenges, care inequities, and a rising demand for timely and personalized interventions and treatments [6]. The demand for services simply does not match those currently available.

Digital innovations like App4Independence (A4i), MindLAMP, and Hope by Centre for Addiction and Mental Health (CAMH) are examples in the mental health field. A4i is an app that was codeveloped with service users to support individuals living with psychosis, offering features such as appointment reminders, medication tracking, and communication with care providers [7-9]. MindLAMP is a platform that enables digital phenotyping by collecting passive and active data to support personalized mental health care and early intervention [10,11]. Hope by CAMH is a digital safety planning intervention embedded in an app for supporting suicide prevention [12,13]. These and other such digital innovations hold the potential to extend the reach of mental health services beyond traditional in-person settings, enable continuous monitoring and early intervention, and support personalized mental health care while attempting to address scalability in ways that traditional clinical service models cannot [14]. Yet, despite this potential, the field remains stuck in a cycle of fragmented, small-scale initiatives that rarely reach full-scale implementation in routine clinical care [1,5,15,16]. Although numerous digital mental health innovations show promise [10,17-19], few are integrated into the realities of mental health clinical care. This disconnect highlights a critical need for more than innovation; infrastructure, sustained commitment, and thoughtful investment are required to integrate digital mental health innovations into routine clinical care. As we examine these needs, the Non-Adoption, Abandonment, Scale-Up, Spread, and Sustainability (NASSS) implementation science framework offers a valuable lens for understanding the complexities that hinder the widespread and long-term uptake or sustainability of digital mental health innovations [20].

## Using Implementation Science to Understand the Current State

The NASSS implementation science framework, which was developed by Greenhalgh and colleagues [20], offers a comprehensive lens for examining why many digital mental health innovations struggle with wide-scale adoption and with being sustainable. The framework highlights the following seven interconnected domains that influence the success and failure of health technologies (inclusive of digital mental health innovations): (1) the condition, (2) the technology, (3) the value proposition, (4) the adopter system, (5) the organization, (6) the wider context (eg, policy), and (7) the dynamic interaction among these domains over time. When applied to digital mental health, these domains present persistent challenges. Mental health conditions are inherently complex and are often chronic and fluctuating, with high rates of comorbidity [21]. Yet, digital tools are frequently designed for singular diagnoses or narrowly defined user needs, failing to capture the clinical reality of patients. On the technology side, many tools are not interoperable with existing health information systems [22], are cumbersome to use, or are not developed with direct input from the clinicians and patients they aim to serve [23]. These usability gaps and poor contextual fit reduce engagement, adoption, and sustainability. Furthermore, the value proposition of digital mental health innovations is often mismatched among those involved in their development and use; developers or investors may prioritize metrics like download rates or short-term engagement, while patients and clinicians may seek tools that meaningfully improve outcomes and fit within clinical care processes. Regarding the adopter system, clinicians and patients are frequently expected to integrate new technologies without adequate training, support, or alignment with existing workflows [24]. Skepticism, digital literacy gaps, limited trust, and added burden further limit uptake [24]. Organizationally, many health care institutions are not structurally prepared to support innovation. They may lack governance frameworks, dedicated personnel, or protected time for staff to explore and integrate new tools, leading to isolated pilots, research studies, or demonstration projects that never scale [25]. In the wider context, policies, regulations [26], and funding models have lagged behind technological advancements. Reimbursement pathways remain unclear, data privacy regulations vary, and national strategies for digital mental health are often fragmented or absent [27].

The success of digital mental health innovations depends on the systems in which they are present, that is, these systems’ ability to evolve and adapt as technologies, user needs, and societal conditions change. Innovations that face complexity across multiple NASSS domains are particularly vulnerable to failure. Even well-intentioned, evidence-based tools are likely to fail in these conditions. Therefore, understanding and addressing the full range of challenges across all NASSS domains is critical to transforming digital mental health from a landscape of “promise” to one that can be scaled and sustained.

Although the NASSS framework provides a structure for understanding barriers to digital mental health innovation, the broader discipline of implementation science offers a set of approaches for addressing these barriers. Strategies derived from implementation science, such as stakeholder/partner engagement, co-design, and many others, are central to the success of digital interventions. Leveraging the diversity of implementation science tools and methods can help ensure that digital innovations move beyond the pilot stage to achieve sustainable integration in complex health systems [28]. By doing so, implementation science not only diagnoses barriers but also actively guides the “how” of embedding, adapting, and scaling digital tools within routine clinical care.

The NASSS framework was selected for its capacity to capture the layers of complexity of digital mental health implementations [29]. Compared to other frameworks, the NASSS framework explicitly considers the evolving and interconnected nature of domains, which are factors that are important in digital mental health innovation. The NASSS framework also uniquely addresses the temporal dimension of implementation, allowing for consideration of how interventions and systems coevolve over time. Given that many digital mental health tools fail not due to lack of efficacy but due to misalignment with health system structures and support processes, the NASSS framework’s holistic and dynamic design makes it particularly well suited for identifying the root causes of implementation challenges in this field.

## Building the Conditions for Sustainable Digital Mental Health in Canada

Structured, strategic infrastructure is critical to moving beyond the pattern of isolated, short-term projects in digital mental health innovation. The Digital Innovation Hub (DIH) at CAMH in Toronto, Canada, is an example of an embedded model for advancing digital mental health innovation. The idea of the DIH came about as a result of the growing potential utility of digital mental health tools and interventions and the lack of infrastructure for supporting meaningful adoption and use. The goal of the DIH is to support the development of scientifically evaluated digital solutions which aim to prevent and support treatment of mental illness, and have the potential for global impact. The DIH was designed to address the real-world challenges present in the context of digital mental health innovation development, use, and scaling, as outlined in the NASSS domains. It operates across four integrated streams of work that collectively support the full life cycle of digital health innovation. Stream 1 focuses on ensuring that digital interventions are scientifically evaluated and aligned with clinical needs. The DIH provides support for usability testing, implementation evaluation, and co-design with clinicians and patients, helping to ensure that tools are clinically meaningful and grounded in evidence. Stream 2 addresses the digital readiness of patients, ensuring that they have the skills and access needed to effectively use digital tools. One example is the coadaptation of a digital mental health literacy program (Digital Outreach for Obtaining Resources and Skills [DOORS] Canada) [30] that was co-designed with input from people with lived experience and integrated into the Collaborative Learning College (formerly Recovery College) [31]. Stream 3 is centered on building clinician competencies in digital mental health. The DIH offers education [32], mentorship, and practical support to clinicians to promote the effective and confident use of digital tools in practice. These include training, co-design workshops, and opportunities to lead or participate in innovation projects. Stream 4 supports the seamless integration of digital technologies into clinical care processes and supports scaling initiatives. One of these initiatives is the further development of the digital navigator role [33] in partnership with those who have championed this role in the past. By embedding itself within the hospital’s operational infrastructure, the DIH facilitates alignment with organizational priorities, streamlines processes for conducting research in this space, and ensures that innovations are adopted and sustained within clinical workflows. One example of work in this space is a close collaboration with the hospital’s Industry Partnerships and Technology Transfer Office to identify ways of supporting commercialization and scaling.

**Figure 1.**
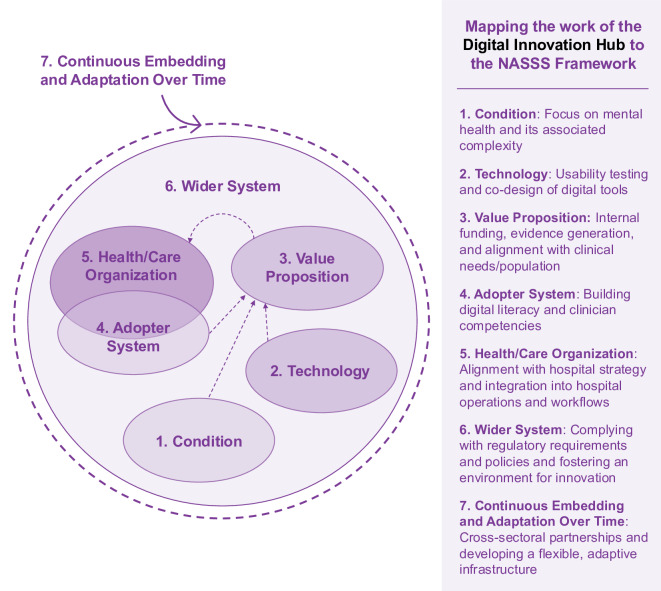
The Digital Innovation Hub and Non-Adoption, Abandonment, Scale-Up, Spread, and Sustainability (NASSS) framework mapped.

The work of the DIH purposefully aligns closely with the NASSS implementation science framework by addressing challenges across all seven domains (Figure 1). The *condition* domain is addressed through a focus on mental health and its associated complexity. The *technology* domain is supported in a number of ways, including through rigorous usability testing and co-design to ensure that digital tools are user-friendly and clinically relevant. With regard to the *value proposition* domain, value proposition is enhanced through internal funding, evidence generation, and alignment with clinical needs. The *adopter system* domain is tackled through dedicated efforts to build digital mental health literacy among patients and competencies among clinicians. The *health organization* domain is engaged through the DIH’s integration into hospital operations, supporting research and innovation within real clinical settings. The *wider system* domain is considered (in one way) through partnerships with industry and technology transfer offices to support commercialization and scale-up. Finally, the *embedding and adaptation over time* domain is addressed by fostering a large, cross-disciplinary community and offering flexible infrastructure that supports continuous iteration and learning. Through the DIH’s infrastructure, which has been developed to map onto the NASSS framework, the hub exemplifies a strategy for supporting sustainable digital mental health innovation.

Although the DIH is still in its early stages (having begun in late 2024), its impact is already evident. A community with over 260 members promotes cross-disciplinary collaboration and knowledge sharing. By operating across the aforementioned four streams and providing infrastructure that supports the full innovation journey, the DIH helps overcome the common pitfalls that hinder digital mental health innovations. It offers a model for embedding, scaling, and sustaining digital mental health innovation, and it does so by bridging evidence, experience, and implementation.

## Global Collaboration Is Necessary

Looking ahead, advancing digital mental health will require greater collaboration across institutions, countries, and disciplines. Rather than working in isolation, organizations can achieve more by intentionally leveraging each other’s infrastructure, expertise, and communities. For example, a meeting was held by teams from several countries (United States, Canada, and Australia) working on the digital navigator role. Examples of how the role has been implemented and evaluated in these countries and lessons learned from these countries were shared among attendees [8,34,35]. This knowledge is essential in the refinement, further development, and scaling of such roles both within countries and internationally.

By working together, we can share implementation knowledge and practical lessons that are aligned with frameworks like the NASSS framework, thereby reducing duplication and accelerating progress. Joint efforts in training, evaluation, and technology development can help address persistent challenges, such as those regarding adoption gaps, digital literacy, and workflow integration. For example, supporting roles like the digital navigator role, which helps bridge the gap between patients, clinicians, and digital tools, can be more widely adopted and refined when multiple partners collaborate. This kind of shared, cross-institutional work does not require uniformity or a one-size-fits-all approach; rather, it enables context-sensitive innovation by aligning on core principles, adapting shared tools to local needs, and managing complexity collectively.

## Conclusion

As the field of digital mental health matures, the challenge is no longer a lack of innovation but a lack of sustained integration into clinical care. The NASSS framework reveals that navigating complexity across multiple domains, from the nature of mental health conditions to the dynamics of organizational change and evolving policy landscapes, is critical to realizing the full potential of digital mental health innovations. Success depends not only on the quality and efficacy of the technology but also on whether the right infrastructure, support systems, and collaborative relationships are in place to embed that technology into everyday routine care. Transforming digital mental health care requires investing in the infrastructure for impact. This means prioritizing people, partnerships, and processes as much as we prioritize the digital platforms themselves. It also means committing to and investing in the “long game” that moves beyond pilots and toward meaningful, measurable, and sustained change. The time to act is now. Implementation science gives us the road map. It is up to us to follow it together.

## Abbreviations

A4i: App4Independence
CAMH: Centre for Addiction and Mental Health
DIH: Digital Innovation Hub
DOORS: Digital Opportunities for Outcomes in Recovery Services
NASSS: Non-Adoption, Abandonment, Scale-Up, Spread, and Sustainability
